# CSF neurogranin levels as a biomarker in Alzheimer’s disease and frontotemporal lobar degeneration: a cross-sectional analysis

**DOI:** 10.1186/s13195-024-01566-w

**Published:** 2024-09-06

**Authors:** Vanesa Jurasova, Ross Andel, Alzbeta Katonova, Katerina Veverova, Terezie Zuntychova, Hana Horakova, Martin Vyhnalek, Tereza Kolarova, Vaclav Matoska, Kaj Blennow, Jakub Hort

**Affiliations:** 1 Memory Clinic, Department of Neurology, Second Faculty of Medicine, Charles University, Motol University Hospital, Prague, Czech Republic; 2 Edson College of Nursing and Health Innovation, Arizona State University, Phoenix, AZ USA; 3 International Clinical Research Center, St. Anne’s University Hospital Brno, Brno, Czech Republic; 4 Department of Clinical Biochemistry, Hematology and Immunology, Homolka Hospital, Prague, Czech Republic; 5 Department of Psychiatry and Neurochemistry, Institute of Neuroscience & Physiology, Sahlgrenska Academy at the University of Gothenburg, Mölndal, Sweden; 6 Clinical Neurochemistry Laboratory, Sahlgrenska University Hospital, Mölndal, Sweden

**Keywords:** Neurogranin, Alzheimer’s disease, Frontotemporal lobar degeneration, Memory, *APOE*

## Abstract

**Background:**

There is initial evidence suggesting that biomarker neurogranin (Ng) may distinguish Alzheimer’s disease (AD) from other neurodegenerative diseases. Therefore, we assessed (a) the discriminant ability of cerebrospinal fluid (CSF) Ng levels to distinguish between AD and frontotemporal lobar degeneration (FTLD) pathology and between different stages within the same disease, (b) the relationship between Ng levels and cognitive performance in both AD and FTLD pathology, and (c) whether CSF Ng levels vary by apolipoprotein E (*APOE*) polymorphism in the AD continuum.

**Methods:**

Participants with subjective cognitive decline (SCD) (*n* = 33), amnestic mild cognitive impairment (aMCI) due to AD (*n* = 109), AD dementia (*n* = 67), MCI due to FTLD (*n* = 25), and FTLD dementia (*n* = 29) were recruited from the Czech Brain Aging Study. One-way analysis of covariance (ANCOVA) assessed Ng levels in diagnostic subgroups. Linear regressions evaluated the relationship between CSF Ng levels, memory scores, and *APOE* polymorphism.

**Results:**

Ng levels were higher in aMCI-AD patients compared to MCI-FTLD (F[1, 134] = 15.16, *p* < .001), and in AD-dementia compared to FTLD-dementia (F[1, 96] = 4.60, *p* = .029). Additionally, Ng levels were higher in FTLD-dementia patients compared to MCI-FTLD (F[1, 54]= 4.35, *p* = .034), lower in SCD participants compared to aMCI-AD (F[1, 142] = 10.72, *p* = .001) and AD-dementia (F[1, 100] = 20.90, *p* < .001), and did not differ between SCD participants and MCI-FTLD (F[1, 58]= 1.02, *p* = .491) or FTLD-dementia (F[1, 62]= 2.27, *p* = .051). The main effect of diagnosis across the diagnostic subgroups on Aβ_1−42_/Ng ratio was significant too (F[4, 263]=, *p* < .001). We found a non-significant association between Ng levels and memory scores overall (β=-0.25, *p* = .154) or in AD diagnostic subgroups, and non-significant differences in this association between overall AD *APOE* ε4 carriers and non-carriers (β=-0.32, *p* = .358).

**Conclusions:**

In this first study to-date to assess MCI and dementia due to AD or FTLD within one study, elevated CSF Ng appears to be an early biomarker of AD-related impairment, but its role as a biomarker appears to diminish after dementia diagnosis, whereby dementia-related underlying processes in AD and FTLD may begin to merge. The Aβ_1−42_/Ng ratio discriminated AD from FTLD patients better than Ng alone. CSF Ng levels were not related to memory in AD or FTLD, suggesting that Ng may be a marker of the biological signs of disease state rather than cognitive deficits.

## Background

Alzheimer’s disease (AD) and frontotemporal lobar degeneration (FTLD) are two of the leading causes of dementia in older adults. AD is a progressive neurodegenerative disease primarily defined by memory loss [1] and characterized by two main histopathological features: extracellular amyloid plaques and intracellular neurofibrillary tangles formed by phospho-tau (p-tau) deposited in brain tissue [2]. FTLD is a clinically, pathologically, and genetically heterogeneous disease that can present with decline in behavior, speech and language, as well as motor and/or psychiatric features [3] and is characterized by the progressive degeneration of the frontal and temporal lobes of the brain [4]. The pathology of FTLD involves several distinct histopathological features, including intraneuronal, filamentous, hyperphosphorylated microtubule-associated protein tau, hyperphosphorylated, ubiquitinated and cleaved RNA- and DNA‐binding protein TDP‐43, or the Fused in Sarcoma (FUS) protein [5].

Much effort has been made in the field of biomarkers to distinguish AD from other neurodegenerative diseases, including FTLD. This differentiation is essential for accurate diagnosis and appropriate treatment planning, progressing in research, and offering assistance to patients and their families in dealing with these conditions. Biomarkers play a critical part in this context by providing concrete metrics to aid in diagnosis and disease surveillance. The loss of neurons and synapses, a prominent feature of progressive neurodegeneration seen in dementia, is accompanied by the deposition of synaptic protein aggregates in the brain, which are eventually released into the cerebrospinal fluid (CSF). Neurogranin (Ng), a postsynaptic protein found in dendritic spines, has been found to be expressed predominantly in the cortex, hippocampus and amygdala [6], brain regions most affected by AD-related neurodegeneration. Therefore, elevated Ng levels in CSF may distinguish AD from other neurodegenerative diseases such as FTLD [7–10], possibly as early as the preclinical stage [11–13]. Early and accurate diagnosis of AD can lead to more timely interventions, potentially assisting in strategies to slow the progression of the disease. The Aβ_1−42_/Ng ratio was studied for a possible improvement in the differential diagnosis of AD too. The Aβ_1−42_/Ng ratio showed better accuracy compared to Ng alone, but was not significantly different from the Aβ_1−42/1−40_ ratio in separating AD dementia patients from non-AD dementia patients [8, 14]. Given its presence in the cortex and the hippocampus, Ng is crucial for long-term potentiation and memory consolidation [15], and elevated levels of Ng in CSF have been associated with poor information transmission and with memory deficits, which tend to present early in AD specifically [16–18]. Currently, there is a lack of specific biomarkers that can reliably distinguish between amnestic mild cognitive impairment (aMCI) due to AD and AD dementia through biological staging of the disease. Differentiating between the two stages of the disease can help in progression monitoring and tailoring treatment strategies specific to the stage of the disease. Accurate biomarkers can aid in the selection and monitoring of participants in clinical trials, leading to more effective drug development. Ng, given its role in synaptic function and memory, shows promise in filling this gap and providing a clearer clinical picture. However, to our knowledge, no study to date has investigated differences in CSF Ng levels across the FTLD diagnostic continuum that would include the pre-dementia, mild cognitive impairment (MCI) stage. Investigating this issue is of great interest because it may reveal early biomarkers that can differentiate between FTLD and AD at the MCI stage, thereby improving diagnostic accuracy and enabling earlier, more targeted interventions. Additionally, validating Ng as a specific biomarker for AD requires comprehensive testing across various pathologies. By investigating Ng levels in FTLD, we not only affirm its role as an AD-specific biomarker but also gain insights into the broader landscape of neurodegeneration. This comparative approach can also enhance our understanding of their underlying mechanisms and might reveal unique pathological features or common pathways across different conditions.

The Apolipoprotein E ε4 (*APOE* ε4) genotype is known to be the strongest genetic risk factor for late-onset AD, with the underlying mechanism of this link being both pre- and postsynaptic dysfunction [19]. Specifically, *APOE* ε4 gene variant promotes Aβ plaque formation [20], which facilitates the loss of key presynaptic proteins [21], as well as disrupts long-term potentiation and plasticity [22] and leads to reduction in dendritic density [23], both indicators of postsynaptic dysfunction. Recent research points to higher levels of Ng specifically in those with MCI and dementia due to AD who are also *APOE* ε4 carriers compared to those without ε4 [14, 24, 25], suggesting an influence of *APOE* in determining Ng levels in the early stages of AD.

Building on prior research, we aimed to investigate whether levels of CSF Ng would be higher in patients with cognitive impairment due to AD—aMCI or AD dementia—vs. cognitive impairment due to FTLD—MCI due to FTLD or FTLD dementia. Then, CSF Ng levels were compared across the diagnostic groups and participants with subjective cognitive decline (SCD). In addition, we also tested the Aβ_1−42_/Ng ratio and its diagnostic accuracy. We also assessed whether higher CSF Ng levels would be associated with worse memory, overall and by diagnostic group, and, using the aMCI due to AD and AD dementia subgroups only, whether *APOE* ε4 status would play a role in any link between CSF Ng and memory. Finally, we tested the same associations with cognitive domains outside of memory including language, visuospatial skills, executive function, and attention and working memory.

## Methods

### Participants

In total, 263 eligible participants were recruited from the Czech Brain Aging Study, a longitudinal, memory clinic-based study on aging and cognitive impairment [26]. All individuals were referred to the clinic by general practitioners, neurologists, or geriatricians based on memory complaints reported by themselves, their relatives, or health professionals.

All of the included participants underwent a spinal tap with CSF collection. Commercial ELISA kits (Innogenetics) were used for dementia biomarker analyses (Aβ_1–42_, total tau [t-tau] and p-tau 181), with cut-off values derived from validation study [26, 27]. In addition to standard clinical and laboratory evaluations, all participants also underwent biochemical analysis of Ng, comprehensive neuropsychological assessment, brain magnetic resonance imaging (MRI), and *APOE* genotyping within three months from the initial visit.

Of the 263 participants, 33 were classified as having SCD [28] and met the published criteria for SCD [28], including a self-reported persistent cognitive decline within the last 5 years in comparison with the previous level which was not related to an acute event, and performance on standardized cognitive tests within the normal range adjusting for age, sex, and education. 14 out of 33 participants with SCD had CSF positive for amyloid biomarkers (reduced Aβ_1–42_). In addition, 109 patients were diagnosed with aMCI due to AD with a high likelihood of AD etiology [29], 67 with AD dementia with evidence of the AD pathophysiological process [30], 25 with MCI due to FTLD (thirteen patients with a behavioural variant of FTD [31], seven patients with a semantic variant of primary progressive aphasia [32], and five patients with a non-fluent variant of primary progressive aphasia [32]) and 29 with FTLD dementia (twelve patients with a behavioural variant of FTD [31], eleven patients with a semantic variant of primary progressive aphasia [32], and six patients with a non‐fluent variant of primary progressive aphasia [32]). Patients with aMCI due to AD and AD dementia had positive CSF AD biomarkers (reduced Aβ_1–42_ and elevated p-tau 181) and patients with MCI due to FTLD and FTLD dementia had CSF negative for amyloid biomarkers (normal Aβ_1–42_). The diagnosis of the MCI stage was based on Petersen’s criteria for MCI [33], with cognitive complaints reported by the patient or caregiver and evidence of cognitive impairment on neuropsychological tests (i.e., cognitive impairment was established when patients scored lower than 1.5 standard deviations below the age- and education-adjusted norms in any cognitive test), as well as generally intact activities of daily living, and no evidence of dementia [33]. In the MCI groups, both MCI single-domain (isolated memory impairment) and MCI multiple-domain (memory impairment plus impairment of at least one other cognitive domain) phenotypes were included. Dementia etiology was diagnosed according to established guidelines, using the Diagnostic and Statistical Manual of Mental Disorders (DSM) V criteria [34, 35].

All participants included in the study signed an informed consent approved by the Motol University Hospital ethics committee.

### Exclusion criteria

The study excluded participants with pre-existing neurological or psychiatric conditions that could impair cognitive function, such as major depressive symptoms, defined as > 8 points on the 15-item Geriatric Depression Scale, stroke, tumor, traumatic brain injury, or multiple sclerosis, hearing difficulties, or significant vascular impairment on the brain MRI (Fazekas > 2) [26].

### Neuropsychological assessment

The neuropsychological battery was composed of the Mini-Mental State Examination (MMSE) as a screening of global cognitive function [36], and the following tests to assess cognitive domains: (1) attention and working memory by Digit Span forward (F-DigitSpan-SC) and backward (B-DigitSpan-SC), adaptation from the Uniform Data Set (UDS-cz 2.0) [37] and Trail Making Test (TMT) A [38], (2) memory by the immediate (LOG-I) and 20-minute delayed (LOG-D) recall of the Logical Memory, adaptation from the Uniform Data Set (UDS-cz 2.0) [37] and Rey-Osterrieth Complex Figure test, reproduction after 3 min (ROCF-R) [39], (3) language by the Boston naming test, 30 odd-items version (BNT-30) [40] and semantic verbal fluency – animals (S-VF-A) [41, 42], (4) executive function by TMT B [38] and phonemic verbal fluency (P-VF; Czech version with letters N-K-P) [41], and (5) visuospatial function by ROCF copy condition (ROCF-C) [39] and the Clock Drawing Test (CDT) [43].

### CSF and serum sample preparation and biochemical analyses

CSF samples were collected and processed in polypropylene 10 mL tubes according to the widely recognized consensus protocol for the standardization of CSF collection and biobanking. Eighteen aliquots of 0.2 ml CSF were stored at − 80 °C in polypropylene tubes for each participant. Blood samples were drawn by venipuncture, allowed to clot at room temperature for 15 min, and then centrifuged at 1700 × g at 20 °C for 5 min within 30 min of collection. Serum supernatant was collected, divided into ten 0.5 ml polypropylene aliquot tubes, and stored at − 80 °C for each participant until further use.

CSF and serum measurements were performed at the Neurochemistry Laboratory at Sahlgrenska University Hospital, Mölndal, Sweden. Following the manufacturer’s instructions, Ng, Aβ_1–42_, Aβ_1–40_, t-tau and p-tau 181 levels in CSF and NfL levels in serum were measured using the LUMIPULSE^®^ G600II instrument (Fujirebio, Ghent, Belgium). It is a cartridge-based system, where monoclonal antibody-coated beads are used for capture and monoclonal antibodies for detection. The resulting luminescence was measured at 477 nm [44].

### *APOE* genotyping

DNA was isolated from whole blood samples (EDTA collection tubes). DNA isolation was performed using Zybio exn3000 isolation system by Zybio Nucleic extraction kit WB-B according to manufacturer protocol. As previously described, *APOE* genotyping was performed according to Idaho-tech protocol (LunaProbes Genotyping Apolipoprotein [*ApoE*] Multiplexed Assay) for high-resolution melting (HRM) analysis [45, 46].

### Statistical analysis

We assessed normality of levels of CSF biomarkers using the Shapiro-Wilk normality test. Because the levels of all of the biomarkers were skewed, we log-transformed the values before conducting main analyses.

Initially we evaluated between-group differences in age, years of education, and scores on cognitive tests including memory using independent samples t-test for differences in means and the Pearson chi-squared test for categorical variables across all subgroups. We also assessed correlations between Ng and Aβ_1-42_, Aβ_1-42/1-40,_ t-tau, p-tau 181 and NfL in patients with AD and FTLD using Pearson’s correlation of log-transformed values of biomarkers adjusted for age and sex, and correlations between Ng levels and MMSE scores adjusted for age, sex and years of education.

Before main analyses, composite scores for cognitive domains were computed by standardizing the raw scores for each neuropsychological test to z-scores using the mean and standard deviation for each subgroup and afterward averaged to create single composite scores for each of the cognitive domains. Before transforming to z-scores, TMT A and B scores, and BNT-30 errors were reversed.

To test our hypotheses, three separate sets of analyses were performed. All of the analyses were done for entire AD and FTLD samples, as well as for all diagnostic subgroups. First, to assess whether Ng levels vary by diagnostic category, we used a one-way analysis of covariance (ANCOVA) that included log-transformed Ng levels as a continuous dependent variable, diagnosis (aMCI due to AD, AD dementia, MCI due to FTLD, and FTLD dementia) as a (categorical) independent variable, with age, sex, and years of education as covariates. Subsequently, Ng levels were also compared in SCD vs. the main diagnostic subgroups.

The magnitude of difference between the diagnostic groups was expressed using eta-squared (η2) value, a measure of effect size commonly reported with analysis of variance, which was then converted to the corresponding value of Cohen’s d [47]. The discriminatory accuracy of CSF Ng was assessed with the receiving operating characteristic (ROC) curve analysis and interpreted with the area under the curve (AUC) values, 95% confidence intervals (CI), and sensitivity and specificity values [48].

Second, to assess if higher Ng levels were associated with worse scores in memory and other cognitive domains across the main diagnostic subgroups—aMCI due to AD, AD dementia, MCI due to FTLD and FTLD dementia—and participants with SCD, we conducted linear regression models. Each model included the averaged cognitive domain z-score as the dependent variable (memory, attention and working memory, language, executive function, and visuospatial function), an interaction between log-transformed CSF Ng levels and diagnosis as an independent variable, and age, sex, and years of education as covariates.

Finally, to assess the role of the *APOE* ε4 genotype and levels of Ng in relation to memory scores, we conducted a linear regression model with memory scores as the dependent variable, Ng levels, *APOE* ε4 carrier status, and their interaction as the independent variable, and age, sex, and years of education as covariates. Given that *APOE* ε4 is a marker of AD, this model was done both for patients with aMCI due to AD and AD dementia, as well as for all of the patients with AD-related etiology.

A two-tailed *p-value* < 0.05 in all analyses was considered statistically significant. Analyses were performed using R statistical language environment.

## Results

### Demographic characteristics

Group demographic and clinical characteristics are reported in Table 1. Participants with aMCI due to AD were significantly older (*p* = .001), carried the *APOE* ε4 allele at significantly higher rates (*p* = .009), and scored significantly worse on BNT-30 (*p* = .017) compared to participants with MCI due to FTLD. On the other hand, individuals with aMCI due to AD scored significantly better on P-VF (*p* < .001) and S-VF-A (*p* = .008) compared to individuals with MCI due to FTLD. Participants with AD dementia were significantly older (*p* = .015), carried the *APOE* ε4 allele at significantly higher rates (*p* < .001), and scored significantly worse on MMSE (*p* = .022), ROCF-R (*p* = .006), ROCF-C (*p* = .014), LOG-I (*p* = .025), and LOG-D (*p* < .001) compared to FTLD dementia participants. Furthermore, individuals with AD dementia scored significantly better on P-VF (*p* = .001).

**Table 1:** Demographic features and cognitive performance of patients with aMCI due to AD, MCI due to FTLD, AD dementia and FTLD dementia

	SCD (*n* = 33)	AD-aMCI (*n* = 109)	AD dementia (*n* = 67)	FTLD-MCI (*n* = 25)	FTLD dementia (*n* = 29)
**Demographics**	Mean ± SD	Mean ± SD	Mean ± SD	Mean ± SD	Mean ± SD
*Age*	68.50 ± 12.03	71.30 ± 8.00 ^d, e^	70.60 ± 8.62 ^d, e^	65.50 ± 6.25 ^b, c,e^	66.00 ± 7.56 ^b, c,d^
*Sex (female/male)*	17/16	66/43	46/21 ^d^	11/14 ^c^	14/15
*Years of education*	15.97 ± 3.11 ^c, e^	14.70 ± 3.28 ^c^	13.60 ± 2.68 ^a, b^	14.40 ± 2.89	14.00 ± 2.68 ^a^
*APOE ε4 carriers*,* n (%)*	27% (9) ^b, c^	53% (58) ^a, d,e^	59% (40) ^a, d,e^	24% (6) ^b, c,e^	27% (8) ^b^,^c, d^
*CSF Neurogranin (pg/mL)*	174.06 ± 61.09 ^b, c^	232.94 ± 96.30 ^a, d^	259.93 ± 109.61 ^a, d,e^	161.45 ± 72.15 ^b, c,e^	208.80 ± 102.56 ^c, d^
*CSF Aβ*_*1−42*_ */Neurogranin ratio*	4.21 ± 1.51 ^b, c^	2.47 ± 1.72 ^a, c,d^	1.88 ± 1.14 ^a, b,e^	5.10 ± 1.39 ^b, e^	4.48 ± 1.39 ^c, d^
*CSF Aβ*_*1−42 *_ *(pg/mL)*	721.70 ± 388.46 ^b, c^	492.51 ± 302.36 ^a, d,e^	426.66 ± 232.11 ^a, d,e^	779.10 ± 321.85 ^b, c,e^	736.15 ± 242.96 ^b, c,d^
*Aβ*_*1−42/1−40 *_ *ratio*	0.07 ± 0.02 ^b, c^	0.05 ± 0.02 ^a, d,e^	0.04 ± 0.01 ^a, d,e^	0.08 ± 0.02 ^b, c,e^	0.07 ± 0.02 ^b, c,d^
*CSF Aβ*_*1−40 *_ *(pg/mL)*	9668.13 ± 3390.06	10086.48 ± 3585.11	9528.48	8601.96 ± 3943.75	8846.86 ± 2713.00
*CSF p-tau 181 (pg/mL)*	38.64 ± 15.39 ^b, c,e^	86.23 ± 54.61 ^a, c,d^	107.67 ± 65.36 ^a, b,d, e^	37.33 ± 17.59 ^b, c,e^	76.20 ± 69.52 ^a, c,d^
*CSF t-tau (pg/mL)*	276.63 ± 101.71 ^b, c,e^	569.06 ± 345.44 ^a, c,d^	712.46 ± 419.80 ^a, b,d^	308.72 ± 141.60 ^b, c^	563.41 ± 419.56 ^a^
*Serum NfL (pg/mL)*	45.40 ± 38.00 ^e^	33.88 ± 27.78 ^e^	41.59 ± 28.81 ^e^	47.94 ± 48.31 ^e^	63.37 ± 51.40 ^a, b,c, d^
**Cognitive Performance**		Mean ± SD	Mean ± SD	Mean ± SD	Mean ± SD
*MMSE score*	29.03 ± 1.00 ^b, c,d, e^	24.80 ± 2.87 ^a, c,e^	18.50 ± 4.12 ^a, b,d, e^	26.00 ± 3.42 ^a, c,e^	21.20 ± 5.93 ^a, b,c, d^
*LOG-I*	14.47 ± 4.58 ^b, c,d, e^	8.69 ± 4.34 ^a, c,e^	4.20 ± 3.33 ^a, b,d, e^	9.74 ± 3.77 ^a, c,e^	6.14 ± 2.80 ^a, b,c, d^
*LOG-D*	13.28 ± 4.43 ^b, c,d, e^	4.96 ± 5.08 ^a, c^	1.16 ± 2.00 ^a, b,d, e^	6.32 ± 5.46 ^a, c,e^	3.90 ± 3.18 ^a, c,d^
*ROCF-R*	15.28 ± 6.62 ^b, c,d, e^	8.49 ± 6.28 ^a, c^	2.47 ± 2.67 ^a, b,d, e^	11.07 ± 6.07 ^a, c,e^	6.66 ± 7.78 ^a, c,d^
*F-DigitSpan-SC*	8.93 ± 1.57 ^b, c,d, e^	8.04 ± 2.12 ^a, c,e^	6.86 ± 2.05 ^a, b^	7.80 ± 2.35 ^a^	6.50 ± 2.55 ^a, b^
*B-DigitSpan-SC*	6.37 ± 1.85 ^b, c,d, e^	5.06 ± 1.95 ^a, c,e^	3.65 ± 1.91 ^a, b,d^	4.76 ± 2.01 ^a, c^	3.92 ± 2.22 ^a, b^
*TMT-A*	44.40 ± 18.08 ^b, c,d, e^	58.50 ± 29.43 ^a, c,e^	107.00 ± 55.91 ^a, b,d^	61.10 ± 32.82 ^a, c^	84.12 ± 59.60 ^a, b^
*TMT-B*	116.42 ± 56.25 ^b, c,d, e^	177.08 ± 81.92 ^a, c,e^	246.04 ± 84.02 ^a, b,d^	183.70 ± 82.22 ^a, c^	234.51 ± 80.18 ^a, b^
*P-VF*	43.5 ± 10.54 ^b, c,d, e^	34.07 ± 13.00 ^a, c,d, e^	24.42 ± 9.82 ^a, b,e^	21.76 ± 10.58 ^a, b,e^	15.39 ± 11.14 ^a, b,c, d^
*BNT-30*	2.93 ± 2.84 ^b, c,d, e^	6.00 ± 4.22 ^a, c,d, e^	10.93 ± 5.96 ^a, b^	8.89 ± 6.04 ^a, b^	11.10 ± 5.89 ^a, b^
*S-VF-A*	23.4 ± 6.16 ^b, c,d, e^	16.24 ± 5.61 ^a, c,d, e^	11.02 ± 5.77 ^a, b^	12.83 ± 5.78 ^a, b^	8.44 ± 5.58 ^a, b^
*CDT*	14.79 ± 1.57 ^b, c,e^	12.69 ± 3.45 ^a, e^	9.31 ± 3.90 ^a, b,d^	14.18 ± 1.70 ^c^	10.74 ± 4.21 ^a, b^
*ROCF-C*	29.29 ± 4.40 ^b, c,e^	26.15 ± 7.30 ^a^	15.33 ± 10.56 ^a, b,d, e^	28.40 ± 6.07 ^c, e^	22.81 ± 10.42 ^a, c,d^

Data are expressed as mean ± standard deviation, except for sex and *APOE* ε4 carrier status. P-values are comparisons using t-test for continuous variables and chi square test for categorical variables

*Abbreviations* AD, Alzheimer’s disease; FTLD, frontotemporal lobar degeneration; aMCI, amnestic mild cognitive impairment; MCI, mild cognitive impairment; SCD, subjective cognitive decline; *APOE*, apolipoprotein E; MMSE, Mini Mental State Examination, total score; LOG-I, Logical Memory Immediate Recall; LOG-D, Logical Memory Delayed Recall; ROCF-R, Rey-Osterrieth Complex Figure, visual reproduction after 3 min; F-DigitSpan-SC, forward Digit Span - score; B-DigitSpan-SC, backward Digit Span - score; TMT-A, Trail Making Test A, given in seconds; TMT-B, given in seconds; P-VF, Phonemic Verbal Fluency; BNT-30, Boston Naming Test, 30 odd-items version, errors after a semantic cue; S-VF-A, Semantic Verbal Fluency - Animals; CDT- Clock Drawing Test, score; ROCF-C, copy condition score

^a^ Indicates statistically significant differences (*p* < .05) between SCD and other diagnostic subgroups

^b^*p* < 0.05 versus AD-aMCI

^c^*p* < 0.05 versus AD dementia

^d^*p* < 0.05 versus FTLD-MCI

^e^*p* < 0.05 versus FTLD dementia

Ng concentrations were significantly higher in patients with AD dementia compared to FTLD dementia (*p* = .035), and in aMCI due to AD compared to MCI due to FTLD (*p* < .001) (see Table 1). Additionally, participants with aMCI due to AD had significantly lower levels of Aβ_1−42_ (*p* < .001) and Aβ_1−42/1−40_ (*p* < .001), and higher levels of p-tau 181 (*p* < .001) and t-tau (*p* < .001) compared to participants with MCI due to FTLD, and participants with AD dementia had significantly lower levels of Aβ_1−42_ (*p* < .001), Aβ_1−42/1−40_ (*p* < .001) and NfL (*p* = .013), and higher levels of p-tau 181 (*p* = .036) compared to participants with FTLD dementia.

Participants with SCD had significantly more years of education than participants with AD dementia (*p* < .001) and FTLD dementia (*p =* .016), carried the *APOE* ε4 allele at significantly lower rates than participants with aMCI due to AD (*p =* .042) and AD dementia (*p =* .003), had significantly higher MMSE, LOG-I, LOG-D, ROCF-R, F-DigitSpan-SC, B-DigitSpan-SC, P-VF, S-VF-A scores compared to all other subgroups (*p* < .001), significantly higher CDT and ROCF-C scores compared to aMCI due to AD, AD dementia, and FTLD dementia (*p* < .001), and significantly lower TMT-A, TMT-B, and BNT-30 scores than all other subgroups (*p* < .001). Ng concentrations were significantly lower in participants with SCD compared to those with aMCI due to AD (*p =* .001) and AD dementia (*p* < .001). Participants with SCD had significantly higher levels of Aβ_1−42_ and Aβ_1−42/1−40_ compared to those with aMCI due to AD and AD dementia (*p* < .001), significantly lower levels of p-tau 181 compared to patients with aMCI due to AD (*p* < .001), AD dementia (*p* < .001), and FTLD dementia (*p =* .005), t-tau compared to those with aMCI due to AD (*p* < .001), AD dementia (*p* < .001), and FTLD dementia (*p* < .001), and NfL compared to FTLD dementia (*p =* .009).

### Ng levels across diagnostic groups

**Fig. 1:**
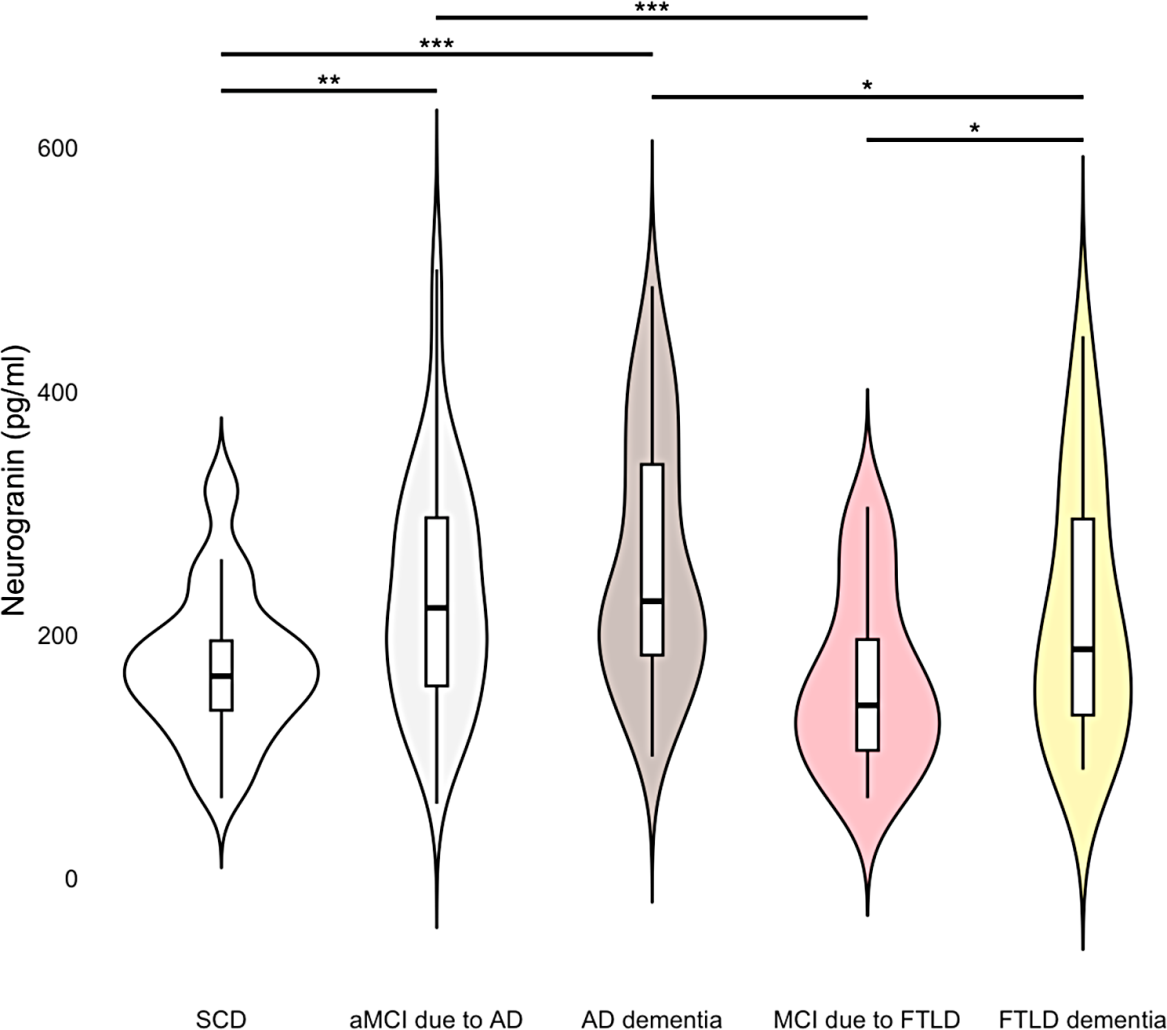
CSF Ng concentrations in subjective cognitive decline (SCD), aMCI due to AD, AD dementia, MCI due to FTLD and FTLD dementia patients. Violin plots showing distribution, box plots, and significant differences assessed by ANCOVA adjusted for age, sex, and education of CSF Ng concentrations. For visual purposes, CSF Ng values are presented raw, not log transformed. *p < .05, **p < .01, ***p < .001

In ANCOVA adjusted for age, sex, and years of education the main effect of diagnosis across the five diagnostic subgroups on Ng levels was significant (F[4, 263] = 8.19, *p* < .001). After performing pairwise comparisons, patients with aMCI due to AD had significantly higher Ng concentrations than patients with MCI due to FTLD (F[1, 134] = 15.16, *p* < .001, η2 = 0.08, AUC = 0.66, 95% CI: 0.62–0.84, with a sensitivity of 0.68 and specificity of 0.72) and those with AD dementia had significantly higher Ng concentrations than those with FTLD dementia (F[1, 96] = 4.60, *p* = .029, η2 = 0.06, AUC = 0.64, 95% CI: 0.49–0.77, with a sensitivity of 0.73 and specificity of 0.52). Ng levels were also higher in patients with AD dementia than in patients with aMCI due to AD although the result only approached statistical significance (F[1, 176] = 2.68, *p* = .067, η2 = 0.02, AUC = 0.58, 95% CI: 0.48–0.67, with a sensitivity of 0.25 and specificity of 0.92). Ng concentrations were also significantly higher in patients with FTLD dementia compared to those with MCI due to FTLD (F [1, 54] = 4.35, *p* = .034, η2 = 0.09, AUC = 0.67, 95% CI: 0.49–0.81, with a sensitivity of 0.72 and specificity of 0.57) (Fig. 1).

Ng levels were significantly lower in patients with SCD than in patients with aMCI due to AD (F[1, 142] = 10.72, *p* = .001, η2 = 0.07, AUC = 0.65, 95% CI: 0.59–0.78, with a sensitivity of 0.62 and specificity of 0.76) or those with AD dementia (F[1, 100] = 20.90, *p* < .001, η2 = 0.19, AUC = 0.75, 95% CI: 0.65–0.84, with a sensitivity of 0.67 and specificity of 0.79). Ng levels were also lower in patients with SCD than in patients with FTLD dementia although the result only approached statistical significance (F [1, 62] = 2.27, *p* = .051, η2 = 0.07, AUC = 0.65, 95% CI: 0.43–0.73, with a sensitivity of 0.45 and specificity of 0.82). Ng levels did not differ between patients with SCD and MCI due to FTLD (F [1, 58] = 1.02, *p* = .491, η2 = 0.01, AUC = 0.56, 95% CI: 0.42–0.73, with a sensitivity of 0.52 and specificity of 0.70).

Additionally, when we stratified the AD sample by *APOE* ε4 carrier status, Ng levels were significantly higher in aMCI due to AD *APOE* ε4 carriers compared to non-carriers (F[1, 109] = 8.22, *p* = .005, η2 = 0.07, AUC = 0.65, 95% CI: 0.57–0.78, with a sensitivity of 0.59 and specificity of 0.69) but not in AD dementia *APOE* ε4 carriers compared to non-carriers (F [1, 67] = 2.20, *p* = .144, η2 = 0.03, AUC = 0.60, 95% CI: 0.49–0.79, with a sensitivity of 0.58 and specificity of 0.33).

In addition, we analyzed the Aβ_1−42_/Ng ratio too. In ANCOVA adjusted for age, sex, and years of education the main effect of diagnosis across the five diagnostic subgroups on Aβ_1−42_/Ng levels was significant (F[4, 263]=, *p* < .001). After performing pairwise comparisons, patients with aMCI due to AD had significantly lower Aβ_1−42_/Ng ratio compared to patients with MCI due to FTLD (F[1, 134] = 41.26, *p* < .001, η2 = 0.23, AUC = 0.78, 95% CI: 0.82–0.94, with a sensitivity of 0.95 and specificity of 0.82), and patients with AD dementia had significantly lower levels of Aβ_1−42_/Ng compared to FTLD dementia too (F[1, 96] = 62.69, *p* < .001, η2 = 0.44, AUC = 0.90, 95% CI: 0.84–0.99, with a sensitivity of 0.89 and specificity of 0.87). Patients with AD dementia had lower levels of Aβ_1−42_/Ng compared to patients aMCI due to AD (F[1, 176] = 7.58, *p* = .007, η2 = 0.04, AUC = 0.61, 95% CI: 0.50–0.68, with a sensitivity of 0.26 and specificity of 0.91). The Aβ_1−42_/Ng ratio differentiated between patients with MCI due to FTLD and FTLD dementia too (F [1, 54] = 4.35, *p* = .045, η2 = 0.10, AUC = 0.68, 95% CI: 0.39–0.76, with a sensitivity of 0.95 and specificity of 0.26).

Participants with SCD had significantly higher levels of Aβ_1−42_/Ng ratio compared to patients with aMCI due to AD (F[1, 142] = 23.95, *p* < .001, η2 = 0.14, AUC = 0.72, 95% CI: 0.71–0.88, with a sensitivity of 0.87 and specificity of 0.69) and AD dementia (F[1, 100] = 71.90, *p* < .001, η2 = 0.40, AUC = 0.88, 95% CI: 0.82–0.96, with a sensitivity of 0.87 and specificity of 0.82). Participants with SCD had only marginally lower levels of Aβ_1−42_/Ng ratio compared to patients with MCI due to FTLD (F [1, 58] = 3.75, *p* = .060, η2 = 0.06, AUC = 0.64, 95% CI: 0.48–0.79, with a sensitivity of 0.43 and specificity of 0.95). There were no statistically significant differences between Aβ_1−42_/Ng ratio in participants with SCD and patients with FTLD dementia (F [1, 62] = 0.00, *p* = .99, η2 = 0.00, AUC = 0.50, 95% CI: 0.38–0.72, with a sensitivity of 0.47 and specificity of 0.74).

### CSF Ng levels and cognitive performance

**Fig. 2:**
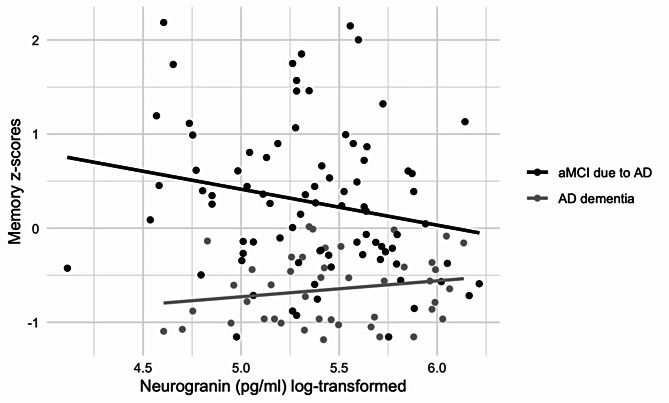
Memory z-scores and Ng levels in aMCI due to AD and AD dementia patients. Memory data are presented as z-scores and Ng data are presented as log-transformed values

The results for the association between CSF Ng levels and performance across cognitive domains are presented in Table 2. Using the entire AD sample, linear regression models adjusted for age, sex, and years of education indicated that Ng levels were negatively, albeit non-significantly, associated with memory scores overall (β=-0.25, *p* = .154), as well as in analyses with aMCI due to AD (β=-0.30, *p* = .147) and positively, albeit non-significantly, with AD dementia (β = 0.20, *p* = .165) (Fig. 2). No significant results emerged for the association between Ng levels and scores on other cognitive domains overall or in diagnosis-specific analyses.

**Table 2:** Association between Ng levels and cognitive domains by diagnosis

	aMCI due to AD and AD dementia			MCI due to FTLD and FTLD dementia			aMCI due to AD			AD dementia			MCI due to FTLD			FTLD dementia	
	Estimate	*p*-value		Estimate	*p*-value		Estimate	*p*-value		Estimate	*p*-value		Estimate	*p*-value		Estimate	*p*-value
Memory	–0.25	0.154		–0.16	0.563		–0.30	0.147		0.20	0.165		–0.10	0.753		0.48	0.173
Language	–0.06	0.714		0.20	0.472		–0.03	0.849		0.54	0.089		0.56	0.141		0.49	0.245
Attention and working memory	–0.19	0.189		–0.17	0.444		–0.04	0.786		–0.16	0.623		–0.14	0.659		–0.07	0.845
Executive function	–0.15	0.383		–0.01	0.965		0.03	0.882		–0.17	0.565		0.10	0.804		0.08	0.800
Visuospatial skills	–0.18	0.340		–0.41	0.141		–0.06	0.731		0.11	0.765		–0.21	0.381		0.09	0.846

*Note* Estimate = unstandardized regression coefficient

**Fig. 3:**
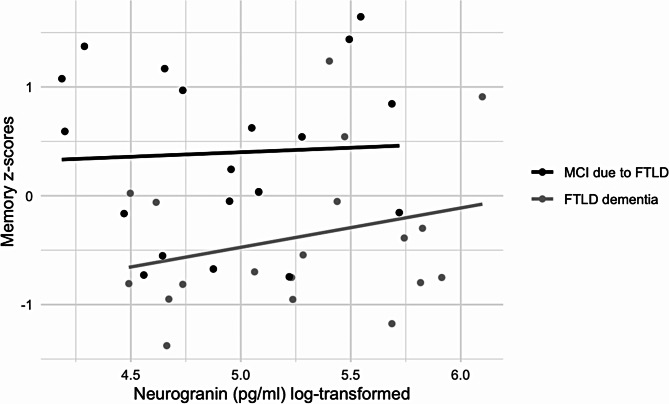
Memory z-scores and Ng levels in MCI due to FTLD and FTLD dementia patients. Memory data are presented as z-scores and Ng data are presented as log-transformed values

Ng levels were again negatively but non-significantly associated with memory scores overall (β=-0.16, *p* = .563) and with MCI due to FTLD (β=-0.10, *p* = .753), and positively but non-significantly with FTLD dementia (β = 0.48, *p* = .173) (Fig. 3). As in AD-related analyses, results for Ng levels and other cognitive domains also did not yield significant results both overall and in analyses conducted by diagnostic group (see Table 2).

Finally, Ng levels were negatively but non-significantly associated with memory scores in participants with SCD (β=-0.78, *p* = .187). As in AD- and FTLD-related analyses, results for Ng levels and other cognitive domains also did not yield significant results in analyses using the SCD subgroup.

Additionally, after adjusting for age, sex, and years of education, Pearson’s correlation analysis revealed that Ng levels did not correlate with MMSE scores in patients with AD (*r*=-.14, *p* = .086) or in patients with FTLD (*r*=-.17, *p* = .273).

### CSF Ng levels and cognitive performance in relation to *APOE* ε4 carrier status in aMCI due to AD and AD dementia

**Fig. 4:**
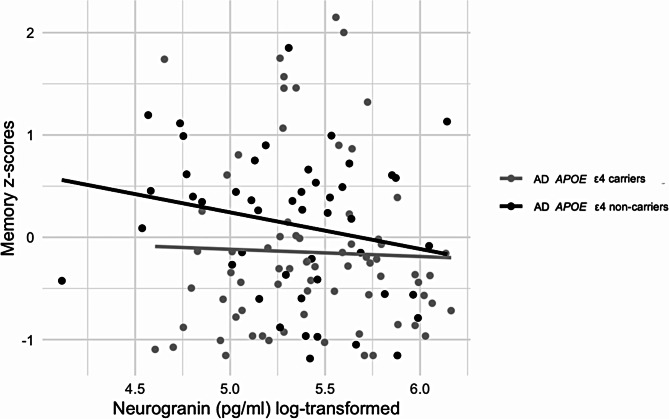
Memory z-scores and Ng levels in *APOE* ε4 carriers and non-carriers with aMCI due to AD and AD dementia. Memory data are presented as z-scores and Ng data are presented as log-transformed values

In linear regression models adjusted for age, sex, and years of education with memory scores as dependent variable and an interaction between Ng levels and *APOE* ε4 carrier status as an independent variable, we found that the association between Ng levels and memory scores did not statistically differ between AD *APOE* ε4 carriers and *APOE* ε4 non-carriers (β=-0.32, *p* = .358) (Fig. 4).

In addition, the association between Ng levels and scores of other cognitive domains did not statistically differ between *APOE* ε4 carriers and *APOE* ε4 non-carriers, including language (β = 0.46, *p* = .181), attention and working memory (β = 0.01, *p* = .980), executive function (β = 0.35, *p* = .335), and visuospatial function (β=-0.11, *p* = .772).

### Correlations of CSF Ng with AD biomarkers

Using the entire AD sample, Ng correlated positively with t-tau (*r* = .77, *p* < .001) and p-tau 181 (*r* = .79, *p* < .001), and negatively with Aβ_1−42/1−40_ (*r*=-.43, *p* < .001). Ng levels did not correlate with Aβ_1−42_ (*r* = .11, *p* = .151) or serum NfL (*r* = .02, *p* = .765). Additionally, in patients with aMCI due to AD, Ng correlated positively with t-tau (*r* = .77, *p* < .001) and p-tau 181 (*r* = .77, *p* < .001), and negatively with Aβ_1−42/1−40_ (*r*=-.52, *p* < .001). Ng levels did not correlate with Aβ_1−42_ (*r* = .12, *p* = .228) and serum NfL (*r* = .15, *p* = .135). In patients with AD dementia, Ng correlated positively with t-tau (*r* = .85, *p* < .001) and p-tau 181 (*r* = .83, *p* < .001), and negatively with Aβ_1−42/1−40_ (*r*=-.53, *p* < .001) and serum NfL (*r*=-.28, *p* = .033) too. Ng levels did not correlate with Aβ_1−42_ (*r* = .16, *p* = .185).

Using the entire FTLD sample, Ng positively correlated with t-tau (*r* = .82, *p* < .001) and p-tau 181 (*r* = .78, *p* < .001), and negatively with Aβ_1−42/1−40_ (*r*=-.51, *p* < .001). Ng did not correlate with Aβ_1−42_ (*r* = .16, *p* = .241) and serum NfL (*r*=-.20, *p* = .161). In subgroup analysis, in patients with MCI due to FTLD, Ng correlated positively with t-tau (*r* = .90, *p* < .001) and p-tau 181 (*r* = .83, *p* < .001), and negatively with Aβ_1−42_ (*r*=-.50, *p* = .010). Ng levels did not correlate with Aβ_1−42/1−40_ (*r*=-.22, *p* = .301) or serum NfL (*r*=-.07, *p* = .727). In patients with FTLD dementia, Ng positively correlated with t-tau (*r* = .82, *p* < .001) and p-tau 181 (*r* = .84, *p* < .001), and negatively with Aβ_1−42/1−40_ (*r*=-.64, *p* < .001) and serum NfL (*r*=-.44, *p* = .018). Ng levels did not correlate with Aβ_1−42_ (*r*=-.05, *p* = .818).

Additionally, using the SCD sample, Ng positively correlated with t-tau (*r* = .76, *p* < .001), p-tau 181 (*r* = .66, *p* < .001), and Aβ_1−42_ (*r* = .68, *p* < .001). Ng did not correlate with Aβ_1−42/1−40_ (*r* = .10, *p* = .590) and serum NfL (*r*=-.17, *p* = .348).

## Discussion

We aimed to investigate the discriminant ability of CSF Ng levels to distinguish between AD and FTLD pathology and between different stages within the same disease, the relationship between Ng levels and cognitive performance in both AD and FTLD pathology, and whether CSF Ng levels vary by apolipoprotein E (*APOE*) polymorphism in the AD continuum. Finally, we tested the role of common CSF biomarkers in our results.

We found that CSF concentrations of the synaptic protein Ng were highest in the group with AD dementia, followed by aMCI due to AD, FTLD dementia, SCD, and MCI due to FTLD, although not all the differences reached statistical significance. We also found that CSF Ng levels were significantly lower in participants with SCD compared to aMCI due to AD and AD dementia, and significantly higher in patients with aMCI due to AD compared to MCI due to FTLD, as well as in patients with AD dementia compared to patients with FTLD dementia. Increased Ng expression in the brain regions affected by AD (i.e. hippocampus and parietal and temporal cortex) could explain the increased Ng levels in the CSF of patients with AD, but not FTLD, as these regions suffer from greater synapse loss than the regions predominantly affected by FTLD [49]. In fact, CSF Ng levels were observably (but non-significantly) higher even in aMCI due to AD compared to FTLD dementia. Our data of higher CSF Ng concentrations in patients with AD are in agreement with previous reports [8, 9, 50–54], as well as with a meta-analysis comparing CSF Ng levels in patients with AD and other neurodegenerative diseases including FTLD [55], highlighting the connection with AD-specific pathophysiological processes that appear to be markedly different from those driving FTLD in the context of markers of postsynaptic dysfunction.

We found that CSF Ng levels were significantly higher in patients with FTLD dementia than in patients with MCI due to FTLD. To our knowledge, this was the first study comparing patients with MCI due to FTLD and FTLD dementia, providing novel information about the potential role of CSF Ng in classifying cognitive status in individuals with FTLD. Either CSF Ng levels increase precipitously over the course of FTLD, or a broader presynaptic dysfunction sets in in relation to FTLD clinical severity that at some point starts to mimic processes underlying AD dementia. Despite the fact that Ng is primarily known to be an AD-specific biomarker, we found that Ng levels were still (marginally, *p* = .051) lower in participants with SCD than in patients with FTLD dementia, although not in MCI due to FTLD (*p* = .491). The difference in SCD vs. FTLD dementia may be due to more widespread neurodegeneration that may encompass structures typically affected in AD dementia.

In terms of disease progression along the AD continuum, increasing levels of CSF Ng have been found to be an indicator of disease stage in several studies [12, 56, 57] and meta-analyses [58, 59], with Ng levels showing reliable increases with greater disease severity, peaking in patients with AD dementia. We could not confirm this finding—although Ng levels were higher in AD dementia compared to aMCI due to AD, the result did not reach statistical significance. The AUC, an indicator of discriminatory accuracy in binary classification models, was 0.58, η2, an indicator of effect size, was 0.02, which is equivalent to Cohen’s d of about 0.29, which signifies a small-to-moderate effect size [47].

The accuracy of the Aβ_1−42/1−40_ ratio in differentiating patients with AD dementia from patients with aMCI due to AD was lower than that of Ng, with AUC 0.52, suggesting that CSF Ng levels have value in evaluating AD-related impairment stage. We also evaluated models adjusted for age, sex, and education. Several relevant studies also found that CSF Ng was unable to statistically distinguish individuals in the AD dementia stage from those with aMCI due to AD [13, 52, 60, 61]. Taken together, Ng appears to have the same discriminatory power between patients with AD dementia and aMCI due to AD as the Aβ_1−42/1−40_ ratio, although the diagnostic accuracy reflects only small-to-moderate effect size.

Additionally, the AUC for differences in Ng levels between FTLD dementia and MCI due to FTLD was 0.67, η^2^ was 0.09, which corresponds to Cohen’s d of 0.63, a medium-to-large effect size. When comparing the magnitude of this effect to the effect obtained from the model contrasting aMCI due to AD against AD dementia (AUC = 0.58, η^2^ = 0.02, Cohen’s d = 0.29), Ng levels seem to be a more pronounced marker of FTLD dementia over MCI due to FTLD than of AD dementia over aMCI due to AD. Overall, CSF Ng levels appear to be equally high in aMCI and dementia stages of AD, but in FTLD, as the disease processes underlying FTLD progress from MCI to dementia stage, elevation of CSF Ng becomes more prominent and similar to AD dementia.

After analyzing the Aβ_1−42_/Ng ratio, we found that the AUC for the differences in Aβ_1−42_/Ng levels between aMCI due to AD and MCI due to FTLD was 0.78, η2 was 0.23, which corresponds to Cohen’s d of 1.09, which is consistent with a large effect size and between AD dementia and FTLD dementia the AUC was 0.90, η2 0.44, and Cohen’s d 1.77, which also corresponds to a large effect size. Compared to Ng alone, the Aβ_1−42_/Ng ratio had a better discriminatory value. When comparing the Aβ_1−42_/Ng ratio between the individual stages of the diseases, we found that the AUC for the differences between aMCI due to AD and AD dementia was 0.61, which represents η2 0.04 and Cohen’s d 0.41, a small-to-medium effect size and between MCI due to FTLD and FTLD dementia was AUC 0.68, η2 0.10 and Cohen’s d 0.67, a medium-to-large effect size. Compared to the stand-alone Ng, we actually saw the same differentiation performances. Being a crucial marker of AD, the Aβ_1−42_ increased the differential performance of Ng between AD and FTLD in both MCI and dementia stages, but more profoundly in the dementia stage with an AUC of 0.90 reflecting an excellent discrimination between AD and FTLD.

Further, we found that in patients with aMCI due to AD and AD dementia combined, Ng correlated positively with t-tau and p-tau 181, as found previously [8, 11, 13, 61–65], and negatively with Aβ_1−42/1−40_ [8, 62]. But we also found that Ng did not correlate significantly with Aβ_1−42_, which also goes along with previous research [11, 13, 61, 63, 64]. In addition, in patients with AD dementia, CSF Ng correlated with serum NfL as well. Additionally, in patients with MCI due to FTLD and FTLD dementia combined, Ng correlated positively with t-tau and p-tau 181 as found previously [66], and negatively with Aβ_1−42/1−40_. We also found that Ng did not correlate with Aβ_1−42_ and serum NfL which also goes with previous research [66]. However, as seen in the AD dementia sample, in our analysis Ng negatively correlated with serum NfL in patients with FTLD dementia too. These results suggest that synaptic degeneration, often indicated by elevated Ng levels in CSF, is associated with tau pathology, neurofibrillary tangle formation, and neurodegeneration in the AD and FTLD continuum.

Higher concentrations of synaptic proteins in CSF presumably reflect the loss of synapses and thus a presumed lower concentration in the brain, as proteins leak into the surrounding fluid. It is known that the Ng expression is highest in the cortex, hippocampus, and amygdala, suggesting a link to cognitive functioning [49]. Ng is directly involved in the long-term potentiation and consolidation of memory traces, where it strengthens long-term potentiation and is related to post-synaptic plasticity [67]. We found no statistically significant associations between Ng levels with memory, language, attention and working memory, executive, or visuospatial domains for all AD, FTLD and SCD groups and for all diagnostic subgroups. Additionally, Ng levels did not correlate with MMSE scores in AD and FTLD groups either. Previously, Headley and colleagues analyzed CSF Ng levels among participants with normal cognition and with MCI due to AD and found that higher Ng levels were associated with lower memory scores in participants with MCI due to AD [16]. Casaletto and colleagues analyzed CSF Ng levels among 132 clinically normal older adults and found that lower Ng concentrations were associated with better performance on delayed recall than those with medium or high Ng concentrations, but CSF Ng was not associated with echoic attention, working memory, or semantic retrieval [18]. Additionally, Rådestig and colleagues analyzed Ng levels among cognitively unimpaired older adults and found that, aside from subtle differences in a few cognitive tests, participants exhibited similar test performance at both high and low levels of CSF Ng [68]. We should note that both Casaletto and colleagues [18] and Rådestig and colleagues [68] studied Ng in relation to individual memory test scores, whereas we used z-scores constructed of multiple tests from the same domain. Therefore, theoretically, our results may reflect real-world associations better.

The effect of *APOE* ε4 genotype on the risk of AD could be explained, at least in part, through direct effects on synaptic function [69]. Although we found that Ng levels were significantly higher in *APOE* ε4 carriers compared to *APOE* ε4 non-carriers among patients with aMCI due to AD, there was no difference in Ng levels between *APOE* ε4 carriers and non-carriers with AD dementia, as found previously [25, 70]. The differences were not confirmed in other studies [71]. However, we found no statistically significant interactions of Ng levels by *APOE* ε4 in relation to memory, language, attention and working memory, executive or visuospatial abilities in the whole AD sample. Therefore, we can assume that *APOE* ε4 carriers may undergo synaptic damage that can be captured by elevated Ng concentrations and that increases the risk of AD as observed through biomarker profile which we examined by an association between Ng and tau pathology, neurofibrillary tangle formation, and neurodegeneration in the AD continuum, notions that should be tested in future research. However, we also found that this increased risk was not necessarily reflected in worse cognitive performance.

This study contributes to the growing body of research on fluid neurodegenerative biomarkers by investigating the utility of Ng in CSF in the diagnosis and stages/progression of AD and FTLD. Our results suggest that CSF Ng levels are elevated in AD compared to FTLD, particularly in the early stages, highlighting its potential as an early diagnostic marker that could complement existing biomarker panels. Furthermore, by examining the Aβ_1−42_/Ng ratio, this study gives more information on more nuanced differential diagnostic strategies. The ability of this ratio to distinguish between AD and FTLD dementia, as seen in our results, could help refine clinical approaches and help target interventions more precisely to individual patients. Ng offers a new perspective as it is associated with synaptic integrity and neuronal activity [72]. Compared to established markers such as tau and Aβ, Ng offers several advantages as a biomarker. While tau and Aβ primarily reflect neurofibrillary tangles and amyloid plaques, Ng provides new insights into synaptic health and neuronal connectivity, which are particularly valuable in identifying disease in its early stages, particularly given that synaptic loss precedes significant neuronal damage [73]. Given our results with Ng, we can speculate that the integration of Ng with other biomarkers such as NfL or glial fibrillary acidic protein (GFAP) can improve the accuracy of differential diagnosis. As mentioned above, Ng is particularly sensitive to early synaptic dysfunction that occurs prior to significant neuronal loss [73]. This makes Ng valuable for early detection and intervention in AD [13]. NfL, a marker for axonal damage and neurodegeneration, and GFAP, a marker for astrocytic activation and gliosis, provide additional information on neurodegeneration and allow a bigger understanding of disease processes. NfL can be elevated in various neurodegenerative and neurological diseases, which limits its specificity in distinguishing between AD and other diseases. On the other hand, NfL levels correlate with disease severity and progression, making it a useful marker for tracking the range of neurodegeneration [74]. GFAP provides insight into the inflammatory and glial responses associated with neurodegenerative diseases and is elevated in several neurodegenerative and non-neurodegenerative neurological diseases. GFAP is more likely to reflect later-stage inflammatory changes, whereas Ng can detect earlier synaptic changes, making Ng more valuable for early diagnosis [75]. Combining Ng with NfL and GFAP together with traditional markers such as Aβ and tau can improve diagnostic accuracy for AD and FTLD by covering different aspects of disease pathology.

Knowledge of the limitations of Ng in predicting cognitive performance also encourages the search for complementary markers and multimodal approaches, including imaging, to fully capture the complexity of neurodegenerative diseases. Imaging techniques such as MRI and PET provide crucial insights into brain structure and function that can be directly correlated with biomarker levels to increase diagnostic accuracy. For example, Massa and colleagues found that the distribution of hypometabolism associated with neuronal loss is distinct from metabolic changes reflecting synapse loss or axonal damage [76].

There are a couple of limitations that need to be noted. First, the cross-sectional design of the study does not allow to assess the potential changes of Ng levels and to track the changes in cognitive performance over time. Additionally, the limited sample size of patients with SCD, MCI due to FTLD and FTLD dementia and different clinical subtypes of FTLD should be taken into consideration before interpreting the data in that the small sample size biases the results towards Type II error, whereby statistical significance was possibly not reached due to lack of power rather than due to the absence of the effect in the real world. Effect sizes, which were often in the moderate range even for non-significant findings, provide some support for this possibility.

Different clinical subtypes of FTLD should be taken into consideration too, although according to the literature there are no significant differences in the Ng levels between behavioural variant of FTD, semantic variant of primary progressive aphasia, and non-fluent variant of primary progressive aphasia subtypes [52]. Although we did not perform this analysis in our study, adding imaging results can be particularly helpful in order to ascertain the role of Ng in FTLD pathology. In addition to the mentioned limitations, it is important to acknowledge the use of serum NfL instead of CSF NfL in our study. Several studies have demonstrated a strong correlation between CSF and serum NfL levels, which supports the reliability of using serum NfL as a proxy for CSF levels [77].

## Conclusions

In conclusion, we found that CSF Ng can be a useful early biomarker of AD-related impairment, although its role as an AD biomarker appears to diminish after dementia diagnosis, whereby the discriminatory value of Ng begins to decrease as the underlying processes related to disease progression in AD and FTLD begin to merge. Being a crucial marker of AD, the Aβ_1−42_ increased the discriminatory role of Ng between AD and FTLD in both MCI and dementia stages, but more so in the post-diagnostic stage. Ng and the Aβ_1−42_/Ng ratio appear to have the same discriminatory power with a small-to-moderate effect size for identifying AD dementia over aMCI due to AD that compares favorably with the discriminatory power of the Aβ_1−42/1−40_ ratio, a common indicator of AD-related pathology. Additionally, we found that Ng levels and the Aβ_1−42_/Ng ratio differ between patients with MCI due to FTLD and FTLD dementia, providing novel information regarding the role of CSF Ng in predicting cognitive status in individuals with FTLD. We also found that *APOE* ε4 carriers may undergo synaptic damage that can be captured by elevated Ng concentrations. Based on our results for the link between CSF Ng and cognitive performance, elevated CSF Ng may be a biomarker of the biological evidence of the disease state rather than a marker of cognitive performance deficits across the cognitive status continuum. Further studies with a longitudinal design and inclusive of imaging markers are needed to confirm current results and further develop Ng-related hypotheses in the context of AD and FTLD diagnosis and progression.

## Data Availability

No datasets were generated or analysed during the current study.

## Abbreviations

Aβ: Amyloid beta
AD: Alzheimer’s disease
aMCI: Amnestic mild cognitive impairment
ANCOVA: Analysis of covariance
APOE: Apolipoprotein
AUC: Area under the curve
B-DigitSpan-SC: Digit Span backward
BNT-30: Boston naming test, 30 odd-items version
CDT: Clock Drawing Test
CSF: Cerebrospinal fluid
F-DigitSpan-SC: Digit Span forward
FTLD: Frontotemporal lobar degeneration
FUS: Fused in Sarcoma
GFAP: Glial fibrillary acidic protein
HRM: High-resolution melting
LOG-D: Delayed recall of the Logical Memory
LOG-I: Immediate recall of the Logical Memory
MCI: Mild cognitive impairment
MMSE: Mini-Mental State Examination
MRI: Magnetic resonance imaging
Ng: Neurogranin
p-tau: Phospho-tau
P-VF: Phonemic verbal fluency
ROC: Receiving operating characteristic
ROCF-C: Rey-Osterrieth Complex Figure test, copy condition
ROCF-R: Rey-Osterrieth Complex Figure test, reproduction after 3 min
SCD: Subjective cognitive decline
S-VF-A: Semantic verbal fluency – animals
TMT: Trail Making Test
t-tau: Total tau
